# PFKP is transcriptionally repressed by BRCA1/ZBRK1 and predicts prognosis in breast cancer

**DOI:** 10.1371/journal.pone.0233750

**Published:** 2020-05-29

**Authors:** Danna Yeerken, Ruoxi Hong, Yan Wang, Ying Gong, Rui Liu, Di Yang, Jinting Li, Jiawen Fan, Jie Chen, Weimin Zhang, Qimin Zhan

**Affiliations:** 1 Key Laboratory of Carcinogenesis and Translational Research (Ministry of Education/Beijing), Laboratory of Molecular Oncology, Peking University Cancer Hospital & Institute, Beijing, China; 2 State Key Laboratory of Oncology in South China, Collaborative Innovation Center for Cancer Medicine, Sun Yat-sen University Cancer Center, Guangzhou, China; CNR, ITALY

## Abstract

**Objectives:**

The present study aims to elucidate the underlying mechanism how PFKP is regulated by BRCA1 and the clinical significance of PFKP in breast cancer.

**Methods:**

MEF-BRCA1^△/△^ and the wild type counterpart MEF-BRCA1^+/+^ cell lines were used to test the sensitivity of glucose depletion in culture medium. Glucose Assay Kit was used to quantify glucose levels in cultural supernatant and cell lysate. Real time PCR was used to measure the mRNA expression levels of genes. Western blot was used to detect protein levels. Chromatin immunoprecipitation was used to verify the bindings between transcription factors and DNA elements. Luciferase reporter assay was performed to determine the transcriptional activity. Histochemistry assay was performed on tissue microarray.

**Results:**

We found that MEF-BRCA1^△/△^ cells consumed more glucose and were more vulnerable to glucose-deprived culture medium. The mRNA profiles and qPCR assay of MEF-BRCA1^△/△^ and MEF-BRCA1^+/+^ cells revealed that PFKP, the rate-limiting enzyme of glycolysis, was significantly upregulated in MEF-BRCA1^△/△^ cells. Consistently, the repressive effects of BRCA1 on PFKP were confirmed by overexpression or knockdown of BRCA1. Moreover, we also demonstrated that PFKP was suppressed by ZBRK1 as well, which was the co-repression partner of BRCA1. Mechanistically, we figured out that BRCA1 formed a transcriptional repression complex with ZBRK1 on the promoter of PFKP and consequently restrained its expression. Importantly, the expression levels of PFKP were demonstrated to associate with poor survival of patients with breast cancer.

**Conclusion:**

Our study provided a new insight into the dysregulation of glycolysis in breast cancer, which might be partially due to the deficiency of BRCA1/ZBRK1 axis and subsequently reversed the transcriptional repressive effect on PFKP. We also found that PFKP overexpressed in a subset of breast cancer patients and could serve as a prognostic factor, which represented a potential target for BC therapy.

## Introduction

Glucose metabolism is the hub of normal operation of the body, providing energy for cell life activities, and coordinating various cellular functions such as gene transcription and epigenetics [1, 2]. However, compared to normal cells, glucose metabolism shifts from the high-capacity oxidized glucose process to a poor efficient aerobic glycolysis type in tumor cells, a phenomenon called "Warburg effect" [3, 4]. Along with the metabolic reprogramming, cancer cells in the uncontrolled proliferation process obtain not only a large amount of energy in such nutrient-poor environment, but also the synthesis of new substances and organelles to meet their nutrition, respiration and tumorigenesis requirements [5–7]. It is worth noting that different tumors have characteristic bioenergetic alterations to benefit their survival and upregulate invasiveness, which means tumor cells are more susceptible and adjustable to this metabolic mode on the other perspective [8, 9]. However, the dynamic and heterogenetic way they influence each other remains to be further understood, which means investigating aerobic glycolysis may provide a new avenue for anticancer therapy.

Phosphofructokinase (PFK) is a major rate-limiting enzyme in glycometabolism process and catalyzes fructose-6-phosphate to convert to fructose-1,6-diphosphate irreversibly [10, 11]. PFK has three types of isoforms and among them PFKP is known as the phosphofructokinase's platelet-specific isoform, which plays vital roles in various types of cancer, including breast cancer [12], glioblastoma [13], renal clear cell carcinoma [14], pancreatic cancer [15], and oral squamous cell carcinoma [16] through metabolic reprogramming. Previous studies have shown that PFKP was closely related with invasion and migration ability of breast cancer cells [17, 18]. What’s more, PFKP was proven to be mainly regulated by the heterogeneity of substrates, and its abnormal expression or structural variation was also one of the critical reasons leading to reprogramming of tumor glycolysis [19]. Furthermore, researches by Nam Hee Kim et al. showed that in the absence of glucose, the transcription factor Snail down-regulated the expression of PFKP, reducing the dependence of tumor cells on glucose and surviving consequently [20]. However, in the case of sufficient supply of energy substances, its mechanism of transcriptional regulation is not yet well-defined.

Recently, Maud Privat and colleagues found that BRCA1 inhibited aerobic glycolysis and promoted an oxidative metabolism to restrict tumorigenesis [21, 22]. Additionally, mounting evidences have indicated that BRCA1 could interact with multifarious metabolic regulators such as, Oct1 [22], p53, Myc, Akt [23] and HIF-1α [24] to mediate metabolic reprogramming of tumor cells. As the rate of mutations in BRCA1 and BRCA2 genes was nearly about 20% among female breast and ovarian cancers, BRCA1 was one of the most significant susceptibility genes in breast cancer [25]. BRCA1 contains C-terminal BRCT domains, which are known as amino-acid sequence motifs, and in N-terminus of the protein, there is a loop finger domain contains E3-ubiquitin ligase activity [26, 27]. BRCA1 works in order by forming complex with Zinc finger and BRCA1-interacting protein with KRAB domain‐1 (ZBRK1) in the promoter region of the gene to regulate gene expression, including *GOT2* [28], *MMP9* [29], *HMGA2* [30], *ANG*1 [31], *and GADD45A* [32] because BRCA1 is unable to recognize the regulatory sequences. ZBRK1 is a representative KRAB-containing zinc finger protein, which participates in regulating transcription of BRCA1's target genes through binding to the canonical motif GGGxxxCAGxxxTTT on their promoters [33]. Thus, it is reasonable to speculate that BRCA1 may participate in transcriptional regulation of the glycolysis-related genes in coordination with ZBRK1.

In this study, we sought to elucidate the molecular mechanism by which BRCA1 affected the reprogramming of glycolysis. We found that deficiency of BRCA1 induced differentiated expression of a subset of glycolytic genes, such as *PFKP*, *ALDOC*, *PGK1* and *ENO3*. Interestingly, we identified that the promoter of PFKP harbored a ZBRK1 consensus DNA-binding element. Subsequently, our findings demonstrated that BRCA1 and ZBRK1 formed a transcriptional repressor complex, which bound to the PFKP promoter and suppressed its expression. Importantly, the clinical relevance study revealed that the expression of PFKP was significantly associated with poor prognosis of patients with breast cancer (BC) and related with different subtypes of BC. Collectively, this study provided a new insight of the transcriptional regulation of PFKP and highlighted the prognostic value of PFKP in BC.

## Materials and methods

### Cell lines and cell culture

Mouse embryonic fibroblast of experimental groups MEF-BRCA1^△/△^ and the wild type control groups MEF-BRCA1^+/+^ (which were kindly supplied by Prof. Chuxia Deng of National Institute of Diabetes, Digestive, and Kidney Diseases), human embryonic kidney cell line HEK293T and human breast cancer cell lines HCC1937, MCF-7 and MDA-MB-231 were cultured in Dulbecco's modified Eagle's medium (DMEM, USA) supplemented with 10% fetal bovine serum (FBS), at 37°C with 5% CO_2_. Glucose-free DMEM with 10% FBS medium was used to maintain MEF BRCA1^+/+^ and MEF-BRCA1^△/△^ cells to detect the sensitivity of these cells upon glucose-deprivation.

### Microarray analysis and ZBRK1 binding motif screening

Expression microarray was performed using mRNA of BRCA1-deficient mouse embryonic fibroblasts MEF-BRCA1^△/△^ and the control counterparts (MEF-BRCA1^+/+^), and the differential gene expression was analyzed to find out candidate genes regulated by BRCA1, especially cellular metabolism related genes. Relative expression fold changes (MEF-BRCA1^△/△^ vs MEF-BRCA1^+/+^) were used to recognize the differentially expressed genes and set fold changes >2 or <0.5 as standard.

The promoter regions of the candidate genes regulated by BRCA1 transcription were further detected according to the data acquired from UCSC Genome Browser (http://genome.ucsc.edu/) to confirm whether the regions contained the ZBRK1 Binding Motif (ZBM) and determine cellular metabolic gene sets regulated by the BRCA1/ZBRK1 transcriptional repression complex.

### RNA isolation and real-time PCR

TRIzol reagent (Invitrogen, USA) was used to isolate the total RNA of breast cancer cells. PrimeScript RT reagent Kit (Promega, USA) was executed to generate the cDNA. Real-time PCR was done by using ABI 7500 real time PCR system (Applied Biosystems, USA). Endogenous expression of GAPDH was used to normalize the expression levels of PFKP mRNA. Primers were designed as below:

GAPDH forward, 5’-TCTCTGCTCCTCCTGTTC-3’,

GAPDH reverse, 5’-GTTGACTCCGACCTTCAC-3’;

PFKP forward, 5’-GGAGTGTGAGTGTGTCCTGG -3’,

PFKP reverse, 5’-ACGCTATGCAGTAGCCTTCC -3’;

### Western blot assay

Cells were collected and lysed in RIPA buffer (Beyotime, China). After quantifying the total protein concentration by Pierce^TM^ BCA protein assay kit (ThermoFisher Scientific, USA), an aggregated amount of 20μg of cellular proteins were separated by 8%–10% SDS/PAGE gel electrophoresis and transferred with 300mA constant current to PVDF membrane. Then 5% bovine serum albumin (BSA) was supplied to block the membrane for an hour after Western blot transfer. Incubated the membrane with primary antibodies, including anti-BRCA1 (CST, USA), anti-ZBRK1 (CST, USA), anti-PFKP (CST, USA), anti-β-actin (Proteintech, China) at 4°C overnight. After primary antibodies washing and secondary antibodies incubation at room temperature for an hour, the chemiluminescence signals were detected with Amersham Imager 600 (GE, USA).

### Glucose consumption assay

Glucose consumption assay was performed by using the Glucose Assay Kit (Abcam, USA) following the manufacturer’s instructions. BRCA1 deficient cells and control groups were cultured in the same volume of DMEM medium with 10% FBS until 70%-80% cell density, then cells were harvested after 12h cultivation in the same volume of fresh DMEM medium with 10% FBS and supplied for protein extraction and quantitation. The cultural supernatants were also collected and quantitated the glucose concentration. The concentration of glucose in fresh medium was also measured. Glucose consumption = amount of glucose in fresh medium–amount of glucose in cultural supernatant, which was normalized by the total protein of each cell line.

### Cell growth glucose dependent assay

Inoculated 500,000 cells in each culture dish and cultured with normal DMEM medium with 10% FBS for 12h. Then cells were cultured with normal DMEM medium with 10% FBS or glucose-free DMEM medium with 10% FBS for another 24h respectively. Each dish randomly selected a field of view to take pictures and counts. After three biological replicates quantified the results.

### Luciferase reporter assay

The 2001bp sequences upstream of initiation codon of PFKP containing the ZBRK1-DNA binding motif was constructed into pGL3-promoter vector (pGL3p). HEK293T was transiently co-transfected of the different plasmid combinations with different concentrations (0, 0.5, 1μg) of BRCA1 or ZBRK1 vector. Then luciferase assay was evaluated by Dual-Luciferase Reporter Assay Kit (Promega, USA) after 48 hours.

### ChIP and ChIP/Re-ChIP assay

Pierce^TM^ Sepharose ChIP kit (ThermoFisher Scientific, USA) was used to carry out ChIP and ChIP/Re-ChIP experiments. After collecting the genomic DNA of the MCF-7 cells, co-immunoprecipitated the DNA fragments with BRCA1 antibody (CST, USA), ZBRK1 antibody (CST, USA) and IgG (as negative control). ChIP/Re-ChIP experiments were performed using BCRA1 antibody for the first round ChIP, then the eluted fractions were incubated with the ZBRK1antibody and ultimately the presence of the PFKP promoter regions in the product was verified by qPCR used the following primers:

PFKP promoter forward, 5’-CCACACTACTCATTAGAAACGCAG -3’,

PFKP promoter reverse, 5’-CACAGTCTTTAGTGATAACCTGCTGA -3’;

### Immunohistochemistry

The expression levels of PFKP were measured by immunohistochemistry (IHC) method in breast cancer tissue and paracancerous tissue microarray which were purchased from SHANGHAI OUTDO BIOTECH CO.LTD. The product number for the tissue microarray was HBre-Duc170Sur-01. The use of the clinical specimens for research purposes was approved by the Institutional Research Ethics Committee of Beijing Cancer Hospital and the Cancer Institute and Hospital (2019KT22). Briefly, sections were routinely dewaxed and rehydrated which were followed by antigen retrieval through immersing in EDTA antigenic recovery buffer and Microwaving. The sections were disposed successively with 3% hydrogen peroxide and 1% goat serum before incubating the tissues with primary antibody overnight at 4°C. Dropwise added biotinylated secondary antibody and used 3, 3’-Diaminobenzidine as coloration after washing.

The scores were calculated by a comprehensive combining of positive staining intensity and tumor cell proportion. The positive staining intensity was divided into scores of 0–3 four levels, which represented negative, weak, moderate and strong. And the proportion of tumor cells was defined as 0 (<5%), 1 (5–25%), 2 (26–50%), 3 (51–75%), 4 (>75%). When analyzing the data, the final scores below 6 were graded as ‘low expression’ (-), otherwise as ‘high expression’ (+), after multiplying the two indicators aforementioned.

### Database clinical data analysis

The correlation between PFKP and ZBRK1 mRNA levels was analyzed by CellMiner database (https://discover.nci.nih.gov/cellminer/). The relationship between breast cancer survival prognosis and PFKP mRNA levels was analyzed by KM plotter database (https://kmplot.com/analysis/).

### Statistical analysis

IBM SPSS Statistics 26 and GraphPad Prism 8 were used for statistical analysis. All data were acquired from at least three independent experiments and were expressed as the mean ±S.D.. Relationship between clinic-pathological parameters and molecular expression was evaluated by two tailed Pearson χ^2^ test. Kaplan-Meier analysis and log-rank test were used for plotting survival curves and comparing the difference. Data were then estimated by multivariate Cox regression. P value was considered statistically significant when below 0.05.

## Results

### Dysfunction of BRCA1 affects cell metabolism

**Fig 1:**
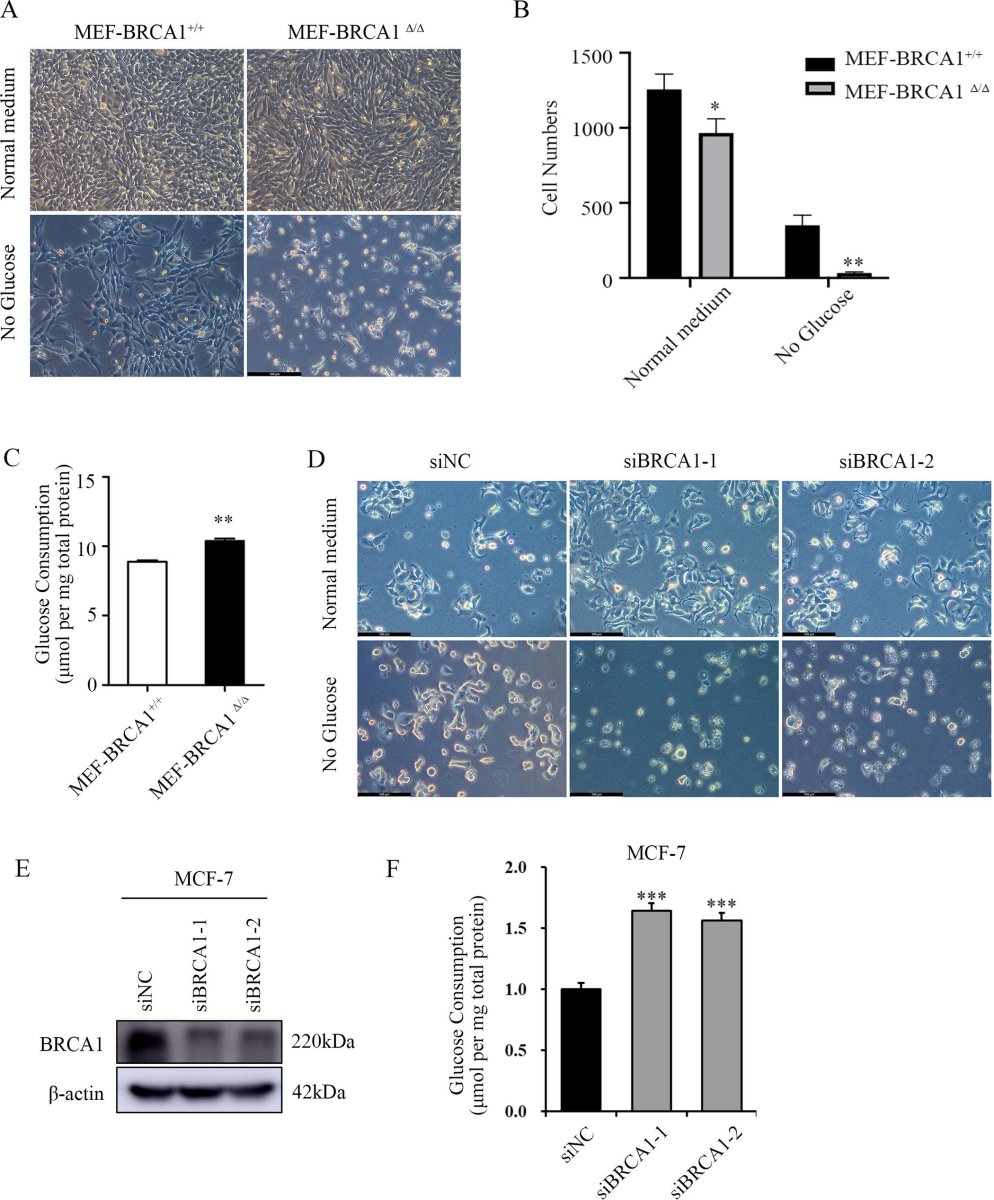
BRCA1 deficient cells are more depend on glucose metabolism. (A) Images represented MEF-BRCA1^△/△^ and MEF-BRCA1^+/+^ cells cultured with normal medium or glucose-deprived medium for 24h. Scale bar: 100μm. (B) Quantification of (A). (C) Glucose consumption of MEF-BRCA1^△/△^ and MEF-BRCA1^+/+^ cells. (D) Images represented BRCA1 deficient MCF-7 cells and control groups were cultured with normal medium or glucose-deprived medium for 24h. Scale bar: 100μm. (E) Western blot examined the expression of BRCA1 in BRCA1 knocked down MCF-7 cells and control groups. (F) Glucose consumption of BRCA1 knocked down MCF-7 cells and control groups. All experiments were biological replications. ***p < 0.001. **P<0.01.

**Fig 2:**
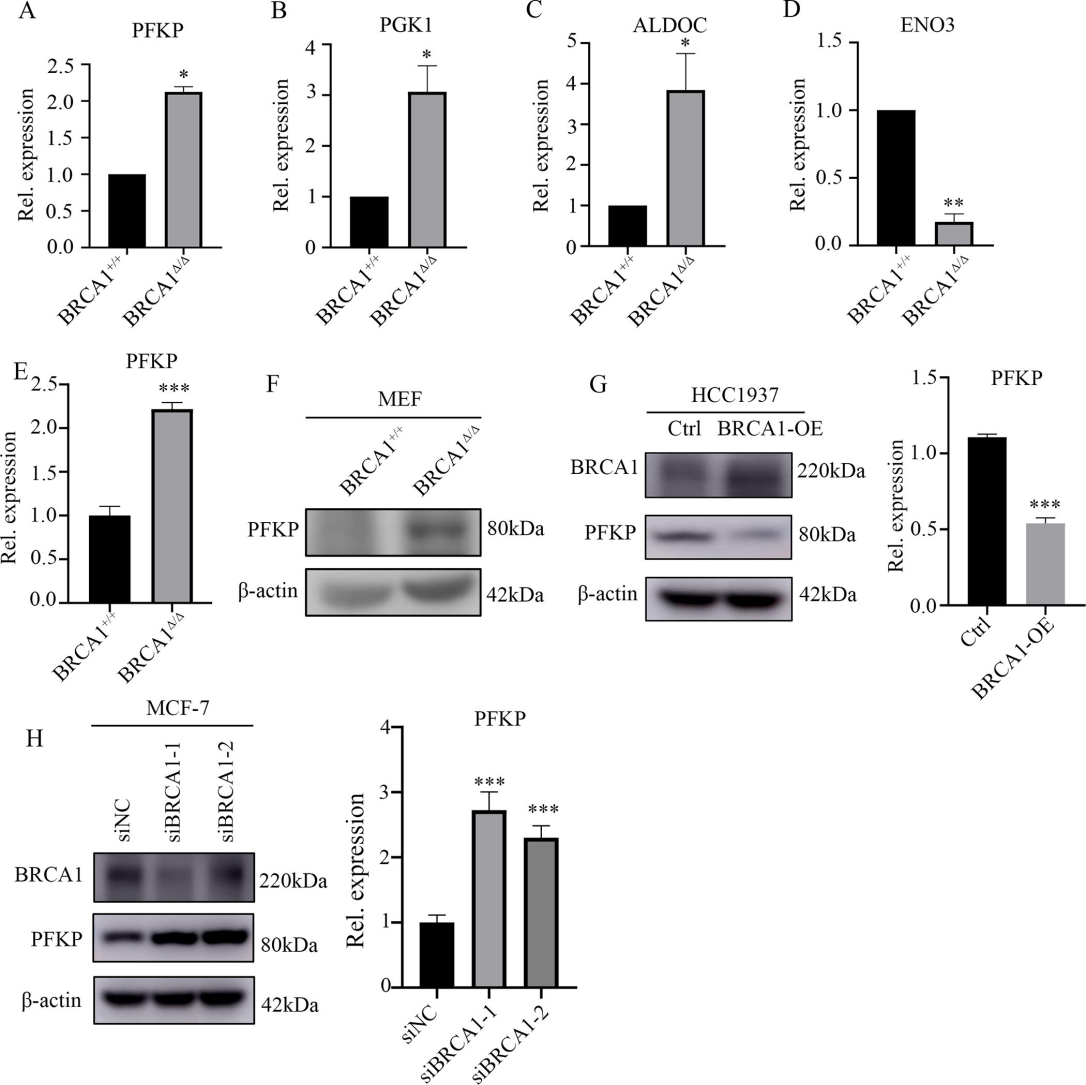
Dysfunction of BRCA1 affects glycolysis related gene PFKP expression. (A-D) The expression level of glycolysis associated genes in BRCA1-deficient mouse embryonic fibroblasts MEF BRCA1^△/△^ cells and wild-type MEF BRCA1^+/+^ cells. (E) Real time PCR confirmed the mRNA level of PFKP in MEF BRCA1^+/+^ cells and MEF BRCA1^△/△^ cells. (F) Western blot verified the protein level in MEF BRCA1^+/+^ cells and MEF BRCA1^△/△^ cells. (G) Western blot and real time PCR detect the expression of PFKP in BRCA1 overexpressed HCC1937 cells and control counterparts. (H) Western blot and real time PCR examined the expression of PFKP in BRCA1 knocked down MCF-7 cells and control groups. All experiments were biological replications. ***p < 0.001. **p < 0.01.*p < 0.05.

To explore the roles of BRCA1 in glucose metabolism, BRCA1 deleted mouse embryonic fibroblast MEF-BRCA1^△/△^ and wild-type MEF-BRCA1^+/+^ were cultured with normal DMEM medium with 10% FBS for 12h. Then cells were cultured with normal DMEM medium with 10% FBS or glucose-free DMEM medium with 10% FBS for another 24h respectively and quantified the results. Interestingly, MEF-BRCA1^△/△^ cells were more vulnerable to glucose deprivation (Fig 1A and 1B). Glucose quantitation assay revealed that MEF-BRCA1^△/△^ cells consumed more glucose than MEF-BRCA1^+/+^cells (Fig 1C). To further verify the relationship between BRCA1 and glucose dependence or consumption in breast cancer cells, we knocked down BRCA1 in MCF-7 and MDA-MB-231 cell lines and repeated above experiments. The results showed that BRCA1 insufficient breast cancer cells were more vulnerable to glucose deprivation (Fig 1D and 1E), and consumed more glucose than their control group (Fig 1F and S1 Fig). To investigate the potential mechanism, we analyzed the expression profiles of mRNA microarray of these paired cell lines. As expected, glycolysis-related genes were significantly altered in MEF-BRCA1^△/△^ cells compared to MEF-BRCA1^+/+^ cells, including *PFKP*, *PGK1* and *ALDOC* were upregulated in BRCA1 deficient cells, while *ENO3* was downregulated (Fig 2A–2D), indicating that BRCA1 participated in the regulation of glycolysis.

Since PFKP is a key rate-limiting enzyme in glycolysis process, we focused on PFKP. To further confirm BRCA1 involvement in PFKP regulation, we first validated the above observations by real time PCR. Indeed, the mRNA level of PFKP was significantly upregulated in MEF-BRCA1^△/△^ cells compared to the wildtypes. Western blotting also estimated that the protein level of PFKP was remarkably increased in BRCA1 deficient cells (Fig 2E and 2F). Furthermore, when we overexpressed BRCA1 in HCC1937 cell line (a BRCA1 deficient breast cancer cell line), there was a notable suppression of PFKP expression in both protein and mRNA level upon BRCA1 overexpression (Fig 2G). Conversely, knockdown of BRCA1 in MCF-7 cell line resulted in the upregulation of the protein and mRNA level of PFKP (Fig 2H). When we repeated above experiments in the MDA-MB-231 breast cancer cell line, the results were consistent (S2 Fig). Taken together, these findings suggested that BRCA1 might inhibit the glycolysis through restraining PFKP.

### BRCA1 binding protein ZBRK1 affects the expression of PFKP

**Fig 3:**
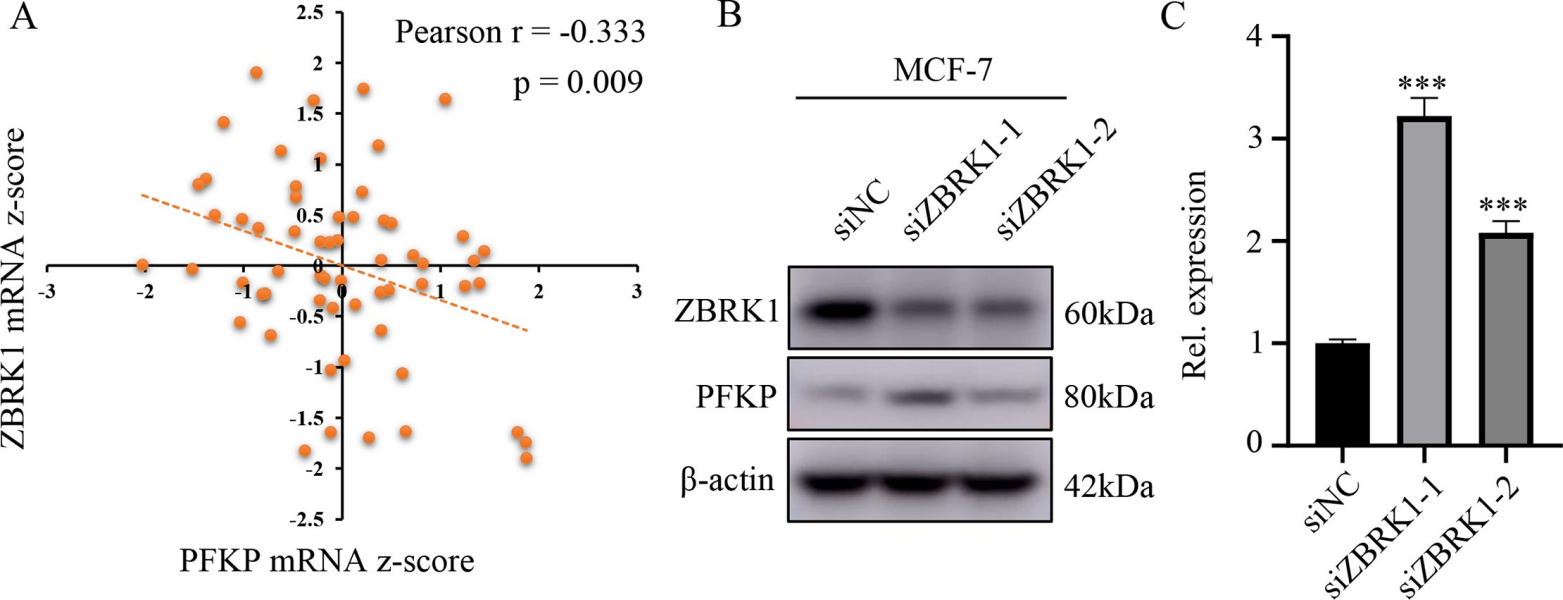
BRCA1 binding protein ZBRK1 affects the expression of PFKP. (A) CellMiner database analysis found that PFKP and ZBRK1 mRNA z-score levels were negatively correlated, Pearson r = -0.333, p = 0.009. (B) PFKP protein levels in ZBRK1 knocked down MCF-7 cells and control cells. (C) PFKP mRNA levels in MCF-7 cells after ZBRK1 knockdown. All experiments were biological replications. ***p < 0.001.

Since BRCA1 bound to the promoter region of the target genes indirectly as reported before, such as *GOT2*, *MMP9*, *HMGA2*, *ANG1* and *GADD45A*, it was well known that BRCA1 generated transcriptional output through binding other transcription factors, and DNA binding protein ZBRK1 usually formed a transcriptional repression complex with BRCA1 to regulate gene expression [28, 34]. Thus, we speculated that BRCA1 exerted transcriptional repression of PFKP by forming a transcriptional repression complex by ZBRK1. Consistent with this notion, by analyzing the sequences of the human PFKP promoter, we found that there were sequences (GGGxxxCAGxxxTTG), which is similar to the canonical ZBRK1-DNA binding motif GGGxxxCAGxxxTTT, located at -599 to -613 upstream of the initiation codons. Additionally, by CellMiner database (https://discover.nci.nih.gov/cellminer/) analysis, we found that the mRNA level of ZBRK1 was moderately negatively correlated with the mRNA level of PFKP among NCI-60 cell lines (Fig 3A). To further verify the regulatory function of ZBRK1 on PFKP, we knocked down ZBRK1 in breast cancer cell line MCF-7 and performed Western blotting and real time PCR assay. The results showed that the mRNA and protein levels of PFKP were increased following the elimination of ZBRK1 (Fig 3B and 3C). Collectively, these findings demonstrated that ZBRK1 was involved in PFKP transcriptional regulation.

### A transcriptional repression complex consists of BRCA1 and ZBRK1 can regulate the expression of PFKP

**Fig 4:**
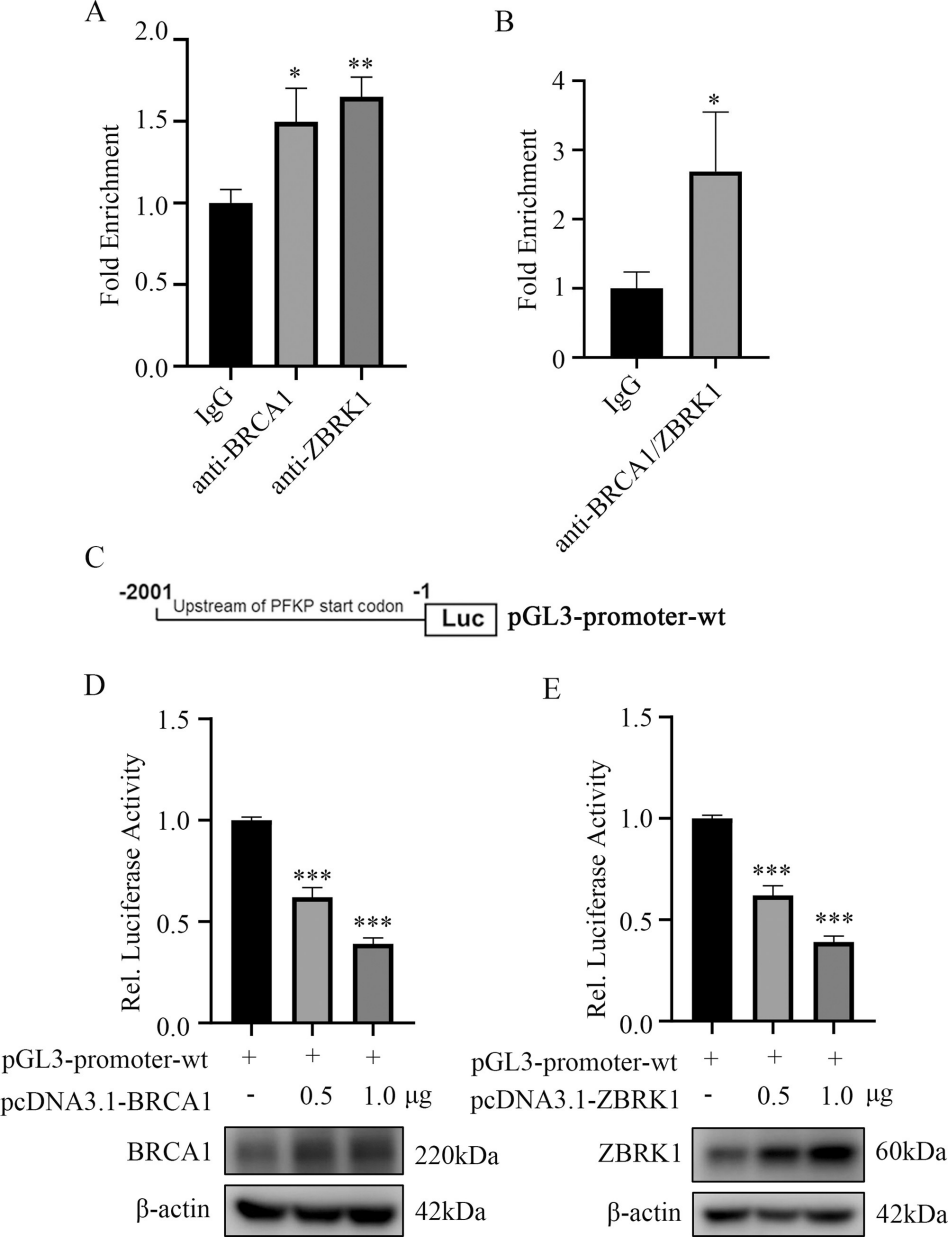
BRCA1 and ZBRK1 form a transcriptional repressor complex and suppress the expression of PFKP. (A) ChIP experiments using BRCA1 and ZBRK1 antibodies to enrich the promoter region of PFKP in MCF-7 cells. Displayed were average real time PCR results and normalized to IgG. (B) ChIP/re-ChIP result in MCF-7 cells with the indicated antibodies. (C) Schematics of PFKP reporter constructs in the promoter region. (D) Relative luciferase activity of reporter constructed with PFKP promoter when different concentrations of BRCA1 plasmids were transfected in HEK293T cells for 48h. (E) Relative luciferase activity of reporter constructed with PFKP promoter when different concentrations of ZBRK1 plasmids were transfected in HEK293T cells for 48h. All experiments were biological replications. ***p < 0.001.**p < 0.01.*p < 0.05.

Given that BRCA1 and ZBRK1 both regulated the expression of PFKP, we sought to elucidate the potential underlying mechanism. It was reasonable to hypothesize that BRCA1 formed a transcriptional repression complex with ZBRK1 to suppress PFKP expression. Accordingly, we first performed chromatin immunoprecipitation (ChIP) and found that the promoter region harbored the potential ZBRK-DNA binding motif was co-immunoprecipitated by anti-BRCA1 and ZBRK1 antibodies, respectively (Fig 4A). Additionally, ChIP/re-ChIP experiments were carried out and revealed that BRCA1 and ZRBK1 formed a transcriptional complex that bound to the promoter region of PFKP (Fig 4B). Collectively, our data revealed a transcription axis that linked BRCA1/ZRBK1 to PFKP.

To further examine the transcriptional ability of BRCA1/ZBRK1 on PFKP, the ZBRK1-DNA binding region of PFKP was constructed into pGL3-promoter plasmid (pGL3-promoter-wt) and the activity of this artificial promoter was analyzed (Fig 4C). By co-transfected pGL3-promoter-wt combined with different concentrations of BRCA1 or ZBRK1 respectively, we found that the transcriptional activity was attenuated upon BRCA1 or ZBRK1 ectopic overexpression, which was indicated by the luciferase activity (Fig 4D and 4E). Taken together, our findings further supported the notion that BRCA1 and ZBRK1 was able to form a transcriptional repression complex to regulate the PFKP transcription.

### The clinical implications of PFKP in patient with breast cancer

**Fig 5:**
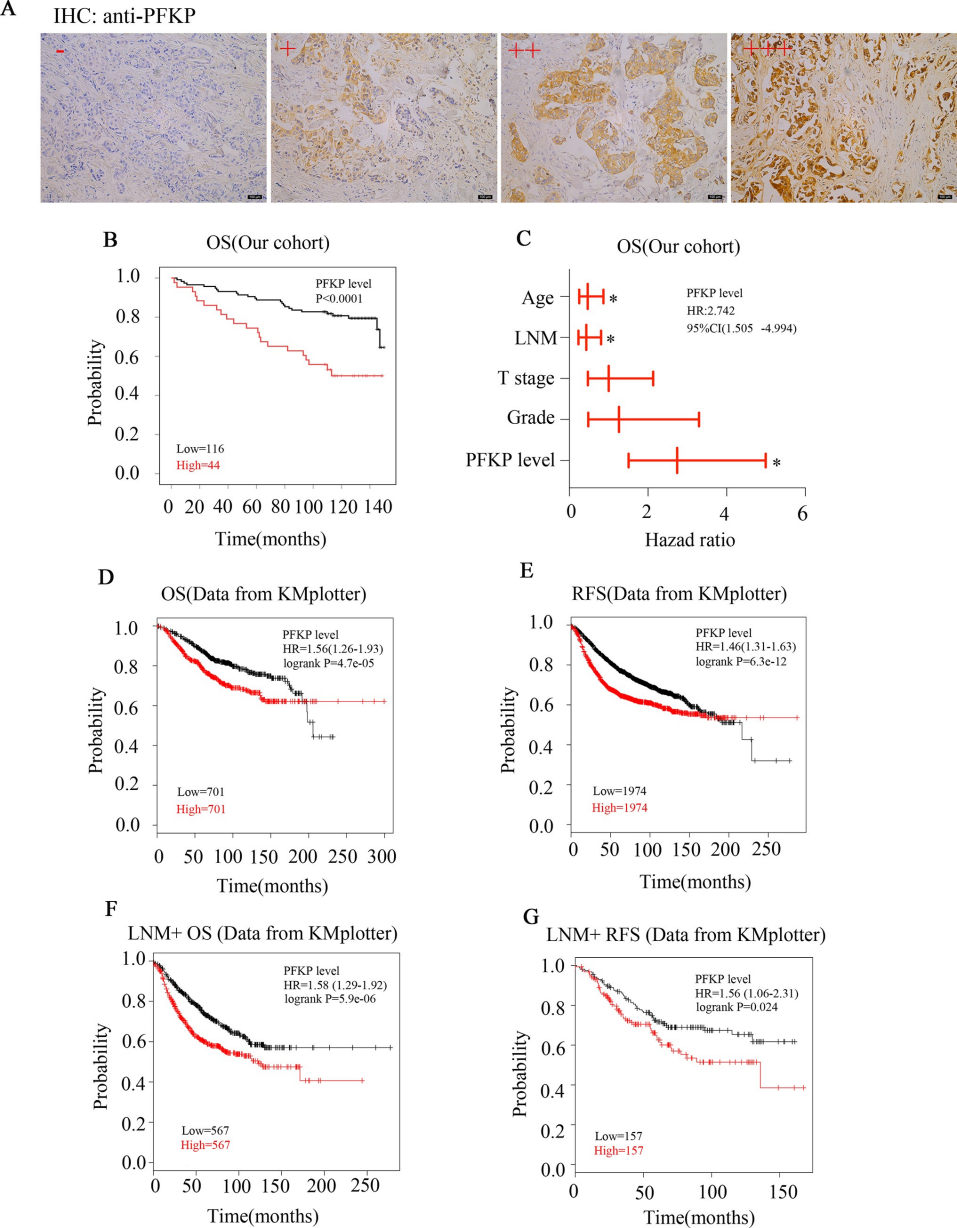
Expression and clinical significance of PFKP in breast cancer. (A) Representative IHC photos of PFKP in breast cancer samples. PFKP expression levels "-" to "+++" mean negative, low positive, moderate positive and high positive. (B) The survival curve of patients with high PFKP levels (n = 44) and low PFKP levels (n = 116) by Kaplan-Meier survival analysis and log-rank test (p<0.0001). (C) Multivariate Cox analysis of breast cancer patients stratified by different classifiers as indicated. (D) KM plotter database showed that PFKP mRNA levels were closely related to overall survival (OS, n = 1402, p = 4.7e-05). (E) KM plotter database showed that PFKP mRNA levels were closely related to relapse free survival (RFS, n = 3948, p = 6.3e-12). (F) KM plotter database showed that PFKP mRNA levels were closely related to overall survival in lymph node metastasis positive (LNM+) patients (OS, n = 1134, p = 5.9e-06). (G) KM plotter database showed that PFKP mRNA levels were closely related to relapse free survival in LNM+ patients (RFS, n = 314, p = 0.024). The expression levels of PFKP in tissue microarray were biological replications. *p < 0.05.

As PFKP was regulated by BRCA1/ZBRK1 axis, we next explored what the role of PFKP in cell growth and colony formation in breast cancer cells MDA-MB-231. The results showed that PFKP deficient decreased the MDA-MB-231 cells ability of growth and colony formation (S3 Fig). To determine the significance of PFKP in patients with breast cancer, we first used immunohistochemistry assay to determine the expression levels of PFKP in tissue microarray containing 160 breast cancer (BC) tissue specimens and 10 adjacent normal tissues (S1 Table). All specimens were invasive ductal carcinoma of female and the different clinicopathological features were summarized in S2 Table. As shown in Fig 5A, 27.5% (44/160) patients showed high PFKP expression while the 10 adjacent normal tissues all represented low PFKP expression, indicating a subset of BC patients carried PFKP overexpression. We then explored the relationship between PFKP levels and different clinic-pathological features using Chi-square analysis. The results revealed that PFKP protein levels were positively related to American Joint Committee on Cancer (AJCC) stage (P = 0.042, Table 1). Interestingly, PFKP protein levels were significantly negatively correlated with hormone receptor expression levels including estrogen receptor and progesterone receptor (ER, P<0.0001and PR, P = 0.001, Table 1, respectively), suggesting patients with hormone receptor negative expression were more prone to possessing PFKP overexpression. Additionally, we also observed that the PFKP protein levels were positively associated with EGFR of cell cytoplasm (EGFR_C, P = 0.011) and EGFR of cell membrane (EGFR_M, P<0.0001, Table 1). Importantly, Kaplan-Meier analysis and log-rank test indicated that high PFKP level was significantly associated poor overall survival of patients with BC (P<0.0001, Fig 5B). Multivariate Cox regression model adjusted by age, lymph node metastasis (LNM), T stage and pathological grade also reported that the level of PFKP could serve as an independent prognostic factor for patients with BC (P = 0.001, HR = 2.742, 95% CI 1.505 to 4.994, Fig 5C and S3 Table).

**Table 1:** Association of PFKP protein level with different clinicopathological features of 160 breast cancer patients.

Clinicopathological features		Total cases (%)	PFKP protein level		*P*‐value
			High (%)	Low (%)	
Age	≤51	82(51.2)	24(29.3)	58(70.7)	0.607
	>51	78(48.8)	20(25.6)	58(74.4)	
Grade	I	18(11.3)	6(33.3)	12(66.7)	0.792
	II	136(85.0)	36(26.5)	100(73.5)	
	III	6(3.7)	2(33.3)	4(66.7)	
AJCC_Stage	1 + 2	107(69.0)	25(22.4)	82(87.6)	**0.042**
	3	48(31.0)	19(39.6)	29(60.4)	
ER_IHC	Positive	102(65.4)	17(16.7)	85(83.3)	**<0.0001**
	Negative	54(34.6)	27(50.0)	27(50.0)	
PR_IHC	Positive	79(51.0)	13(16.5)	66(83.5)	**0.001**
	Negative	76(49.0)	31(40.8)	45(59.2)	
EGFR_C	<6	136(85.0)	32(23.5)	104(76.5)	**0.011**
	≥6	24(15.0)	12(50.0)	12(50.0)	
EGFR_M	<6	144(90.0)	32(22.2)	112(77.8)	**<0.0001**
	≥6	16(10.0)	12(75.0)	4(25.0)	
T_Stage	T1	36(22.8)	9(25.0)	27(75.0)	0.662
	T2+T3	122(77.2)	35(28.7)	87(71.3)	
Lymph node metastasis	YES	45(28.8)	16(35.6)	29(64.4)	0.200
	NO	111(71.2)	28(25.2)	83(74.8)	
Location	Right	89(55.6)	21(23.6)	68(76.4)	0.217
	Left	71(44.4)	23(32.4)	48(68.6)	

To further confirm the clinical importance of PFKP, we next used KM plotter database [35] to analyze the implications of PFKP mRNA levels in prognosis of BC patients. The results showed that PFKP mRNA levels were negatively related to not only overall survival (OS) (P<0.05, HR = 1.56, 95% CI 1.26–1.93, Fig 5D), but also relapse free survival (RFS) (p<0.05, HR = 1.46, 95% CI 1.31–1.63, Fig 5E). We next explored the relationship between PFKP mRNA levels and OS or RFS in lymph node metastasis positive (LNM+) patients by KM plotter database. The results also indicated that PFKP expression was significantly associated with the OS and RFS of LNM+ patients (P<0.05, HR = 1.58, 95% CI 1.29–1.92 for OS and P<0.05, HR = 1.56, 95% CI 1.06–2.31 for RFS, K-M survival analysis and log-rank test, Fig 5F and 5G). Furthermore, we explored the relationship between PFKP mRNA levels and OS or RFS in triple-negative breast cancer patients by KM plotter database. The results suggested that PFKP expression was significantly associated with the OS of triple-negative breast cancer patients, but there was no statistically difference in triple-negative breast cancer patients' RFS (P = 0.047, HR = 2.04, 95% CI 0.99–4.18 for OS, S4 Fig). Collectively, these findings indicated that the expression levels of both protein and mRNA of PFKP were promising prognostic factors for clinical practice of breast cancer.

## Discussion

BRCA1 has been implicated in glycolysis, however, the underlying mechanism is still largely unknown. The rate-limiting enzyme PFKP was previously reported dysregulated in different cancers and the clinical significance of PFKP has been involved in various cancers, however, the clinical association of PFKP in breast cancer was still unclear. In the present study, we confirmed that BRCA1 deficiency was able to induce glycolysis remodeling, which led to increased PFKP expression and more consumption of glucose. Mechanically, our study provided evidences that BRCA1 bound to the promoter of PFKP with the transcriptional repressor ZBRK1 and transcriptionally suppressed the expression of PFKP. Importantly, we also observed that the protein levels of PFKP were significantly associated with poor clinical outcomes and might serve as an independent prognostic factor of patients with BC.

In the previous studies, BRCA1 has been linked to cell metabolism. Under the condition of insufficient BRCA1, fatty acid oxidation and glucose levels were reduced in cardiomyocytes and fatty acid synthesis were decreased in skeletal muscle [36, 37]. Mutations of BRCA1 gene led to metabolic reprograming to meet nutrition requirements of breast epithelial cells through increasing tricarboxylic acid cycle and lipogenesis [38]. Furthermore, glutaminolysis and pentose phosphate pathways were upregulated when BRCA1 was depleted in ovarian cancer [39]. Although it has been demonstrated that BRCA1 played a prominent role in cell metabolic reprogramming, there was no sufficient evidence to support the function and mechanism by which BRCA1 regulated glycolysis in tumor metabolism by means of transcriptional molecule. After microarray analysis of BRCA1 wildtype mouse embryonic fibroblasts MEF BRCA1^+/+^ and BRCA1 knockout mouse embryonic fibroblasts MEF-BRCA1^△/△^, we demonstrated that PFKP was upregulated upon BRCA1 deficiency. PFKP is the “gatekeeper” in glycometabolism process and plays important roles in metabolic reprogramming. To illustrate the relationship between BRCA1 and PFKP, we did comprehensive studies. Firstly, we proved that both BRCA1 and ZBRK1 could inhibit the expression of PFKP through western blot and qPCR experiments. Secondly, the physical occupancy of BRCA1/ZBRK1 within the PFKP promoter region was verified by ChIP and ChIP/re-ChIP assays. Thirdly, luciferase reporter assay further supported that BRCA1/ZBRK complex inhibited PFKP transcription. All works have been done under normoxic condition. In conclusion, our data have illustrated that BRCA1 and ZRBK1 regulated PFKP in transcriptional level by forming a complex on PFKP promoter.

The most recent research showed that AKT activation, which was caused by deficiency of phosphatase and tensin homologue (PTEN) and activation of epidermal growth factor receptor (EGFR)-dependent PI3K, increased PFKP expression by directly binding to PFKP to form a complex and inhibit the proteasomal degradation of PFKP [13]. Our findings were also consistent with this observation that PFKP was positively associated with cytoplasm-and membrane-located EGFR, further confirming the relationship between PFKP and EGFR. At the same time, deficiency of BRCA1 or mutation of BRCA1 gene was proven to activate the AKT oncogenic pathway [40], and BRCA1 was implicated as the pivotal transcriptional regulator of EGFR [41]. Our current findings demonstrated that BRCA1 could regulate PFKP by forming a complex with ZBRK1, so whether BRCA1 comes to play through BRCA1-AKT-PFKP axis or whether the connection between BRCA1 and EGFR play an indispensable role will need further investigation.

Moreover, PFKP was closely associated not only with glycolysis, but epithelial-mesenchymal transition (EMT) under metabolic stress condition through Snail-PFKP axis [16, 20]. At the same time, overexpression of BRCA1 was proved to play an essential role in the process of EMT through EMT markers such as Snail [42, 43]. Given that we have illustrated that dysfunction of BRCA1 or ZBRK1 led to the overexpression of PFKP and subsequently enhanced aerobic glycolysis and the malignant phenotype of breast cancer, it is worthy to elucidate the underlying mechanism that how BRCA1 exerts its functions in regulating the balance of EMT and rapid aerobic glycolysis to meet different situations. At present, our data preliminarily indicated that BRCA1 and Snail might not be related (S5 Fig), but needs further proof.

In previous reports, PFKP was found to be associated with malignancy of some types of tumors, including breast cancer [17, 20, 44], which was further confirmed by our findings that PFKP was overexpressed in a subset of breast cancer tissues. Simultaneously, bioinformatics analysis demonstrated that patients with overexpressed PFKP had poor prognosis, suggesting that PFKP might be used as a survival prognostic factor clinically. Since PFKP was upregulated in breast cancer and negatively associated with the prognostic of breast cancer patients, it had the potential to be a drug target [17, 45]. It has been previously demonstrated that PFK inhibitor clotrimazole (CTZ) effectively compromised the ability of voltage-dependent anion channel 2 (VDAC2) to induce glioma stem cells phenotypic transition and metabolic phenotypic transition [46]. Furthermore, when PFKP inhibitor 2,5-anhydro-D-glucitol-6-phosphate was employed in T24 bladder cancer cells, the cell growth was significantly decreased via suppression of glycolysis [47]. However, PFKP inhibitors have not been fully developed. At the present study, we analyzed by CellMiner that the expression level of PFKP was positively related to the drug activity of protein kinase C (PKC) inhibitor Staurosporine (S4 Table), suggesting that the expression level of PFKP could affect the drug sensitivity of breast cancer cells to PKC inhibitors. Staurosporine is a potent PKC inhibitor currently in clinical phase III trials, which was proven to be associated with BRCA1 expression [48]. In addition, Peroxisome proliferator-activated receptor gamma (PPARγ) was overexpressed in BRCA1-defective breast cancer patients, and in vascular smooth muscle cells PPARγ was found to regulate PFKP [49, 50]. PPARγ activators are commonly used in the treatment of diabetes and obesity since PPARγ is involved in lipid biosynthesis and adipogenesis regulation [51]. Therefore, maybe there are functional connections between BRCA1, PPARγ and PFKP in glycolysis system and PFKP inhibitor may have a potential effect on BRCA1 dysfunctional breast cancer patients who suffer from diabetes and need treatments with PPARγ agonists.

In summary, our results indicate that BRCA1/ZBRK1 affects the transcriptional regulation of PFKP, providing important evidence for the understanding of abnormal mechanism of breast cancer glycolysis. Meanwhile, overexpression of PFKP may serve as an independent poor prognostic factor for patients with breast cancer, which provides an important basis for targeting PFKP in breast cancer patients with BRCA1 or ZBRK1 deficiency.

## Supporting information

S1 Table The clinicopathological characteristics of 160 breast cancer samples and 10 adjacent normal tissues. Detail information of 160 breast cancer samples and 10 adjacent normal tissues analyzed in the article.
(XLSX)

S2 Table Different clinicopathological features of 160 breast cancer patients. Induction of 160 breast cancer samples' characteristics information.
(XLSX)

S3 Table Multivariate COX regression analysis for the identification of prognostic factors for overall survival in BC patients. PFKP protein level, age, distant lymph node metastasis (LNM), T stage, and histopathological grade were included for multivariate Cox regression survival analysis Among 160 breast cancer patients.
(XLSX)

S4 Table Correlations between the PFKP mRNA level and drug activities. The correlation between PFKP expression level and drug activity by CellMiner.
(XLSX)

S1 Fig BRCA1 deficient breast cancer cells are more depend on glucose metabolism. (A) Images represented BRCA1 deficient MDA-MB-231 cells and control groups were cultured with normal medium or glucose-deprived medium for 24h. (B) Glucose consumption of BRCA1 knocked down MDA-MB-231 cells and control groups. All experiments were biological replications. ***p < 0.001.
(TIF)

S2 Fig Dysfunction of BRCA1 or ZBRK1 affects PFKP expression in breast cancer cells. (A) Western blot detected the expression of PFKP in BRCA1 knocked down MDA-MB-231 cells and control counterparts. (B) Real time PCR detected the expression of PFKP in BRCA1 knocked down MDA-MB-231 cells and control counterparts. (C) Western blot examined the expression of PFKP in ZBRK1 knocked down MDA-MB-231 cells and control groups. (D) Real time PCR verified the expression of PFKP in ZBRK1 knocked down MDA-MB-231 cells and control groups. All experiments were biological replications. ***p < 0.001. **p < 0.01.
(TIF)

S3 Fig Dysfunction of PFKP affects the growth of breast cancer cells. (A) MTT assay for growth curve of MDA-MB-231 cells. (B) Colony formation assay in MDA-MB-231 cells showed that PFKP sufficient cells had stronger growth energy. ***p < 0.001. **p < 0.01.
(TIF)

S4 Fig Expression and clinical significance of PFKP in breast cancer. (A) KM plotter database showed that PFKP mRNA levels were closely related to overall survival in triple-negative breast cancer patients (OS, n = 126, P = 0.047). (B) KM plotter database showed that PFKP mRNA levels were not statistically corelated with triple-negative breast cancer patients' relapse free survival (RFS, n = 124, p = 0.2).
(TIF)

S5 Fig The expression level of Snail may not associate with BRCA1. Western blot examined the expression level of Snail in MEF-BRCA1^△/△^ and MEF-BRCA1^+/+^ cells.
(TIF)

S1 Raw Images (PDF)

## Abbreviations

BC: Breast cancer
BRCA1: breast cancer 1
ZBRK1: Zinc finger and BRCA1-interacting protein with a KRAB domain 1
PFKP: ATP-dependent 6-phosphofructokinase, platelet type
ChIP: Chromatin immunoprecipitation‎
AJCC: American Joint Committee on Cancer
LNM: lymph node metastasis
IHC: Immunohistochemistry
