# Supplementary figures and images for: PFKP is transcriptionally repressed by BRCA1/ZBRK1 and predicts prognosis in breast cancer
Source: PLoS One. 2020 May 29;15(5):e0233750. doi: 10.1371/journal.pone.0233750 (PMC7259711; doi:10.1371/journal.pone.0233750)

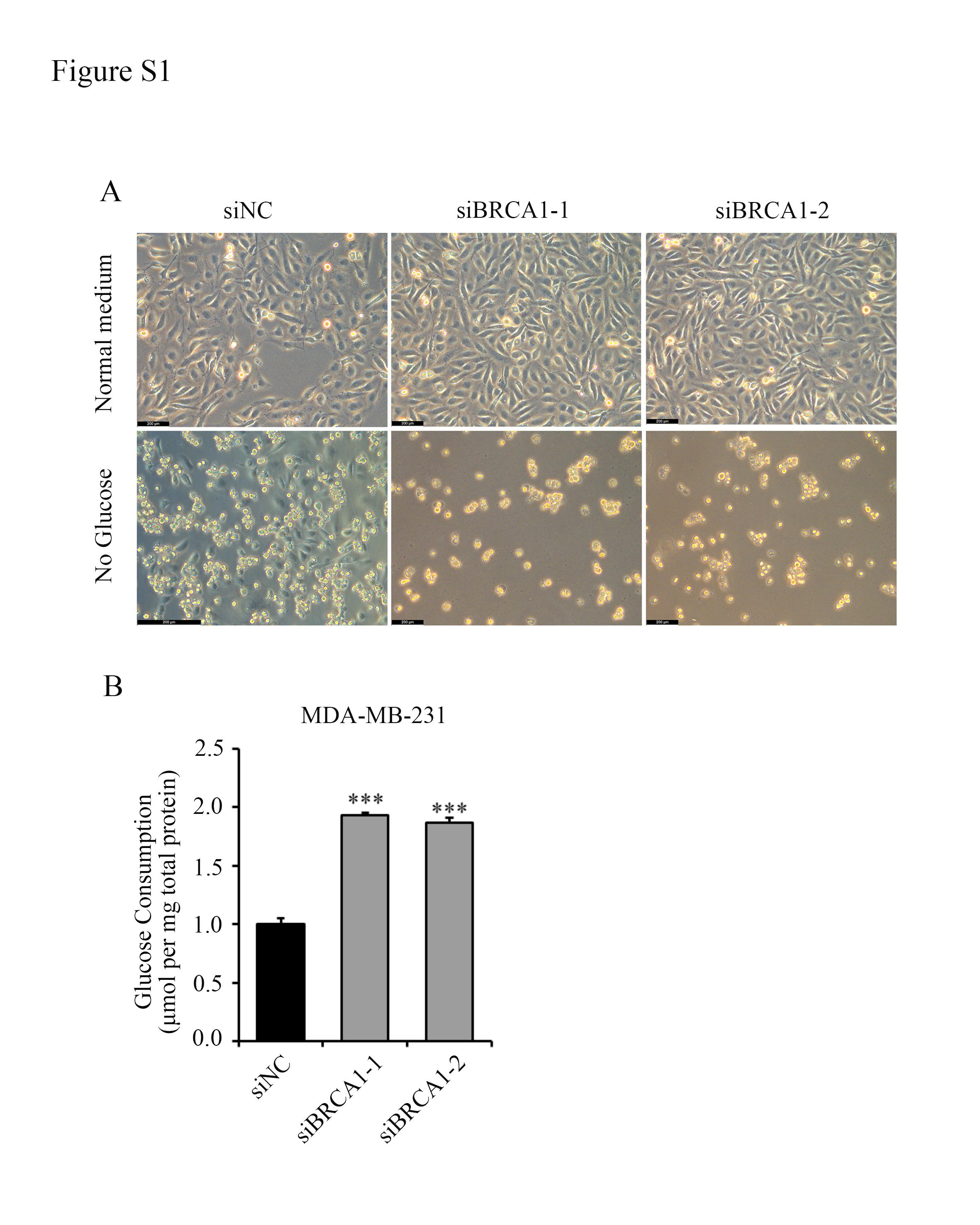

Supplement: S1 Fig — (A) Images represented BRCA1 deficient MDA-MB-231 cells and control groups were cultured with normal medium or glucose-deprived medium for 24h. (B) Glucose consumption of BRCA1 knocked down MDA-MB-231 cells and control groups. All experiments were biological replications. ***p < 0.001. (TIF) [file pone.0233750.s005.tif]

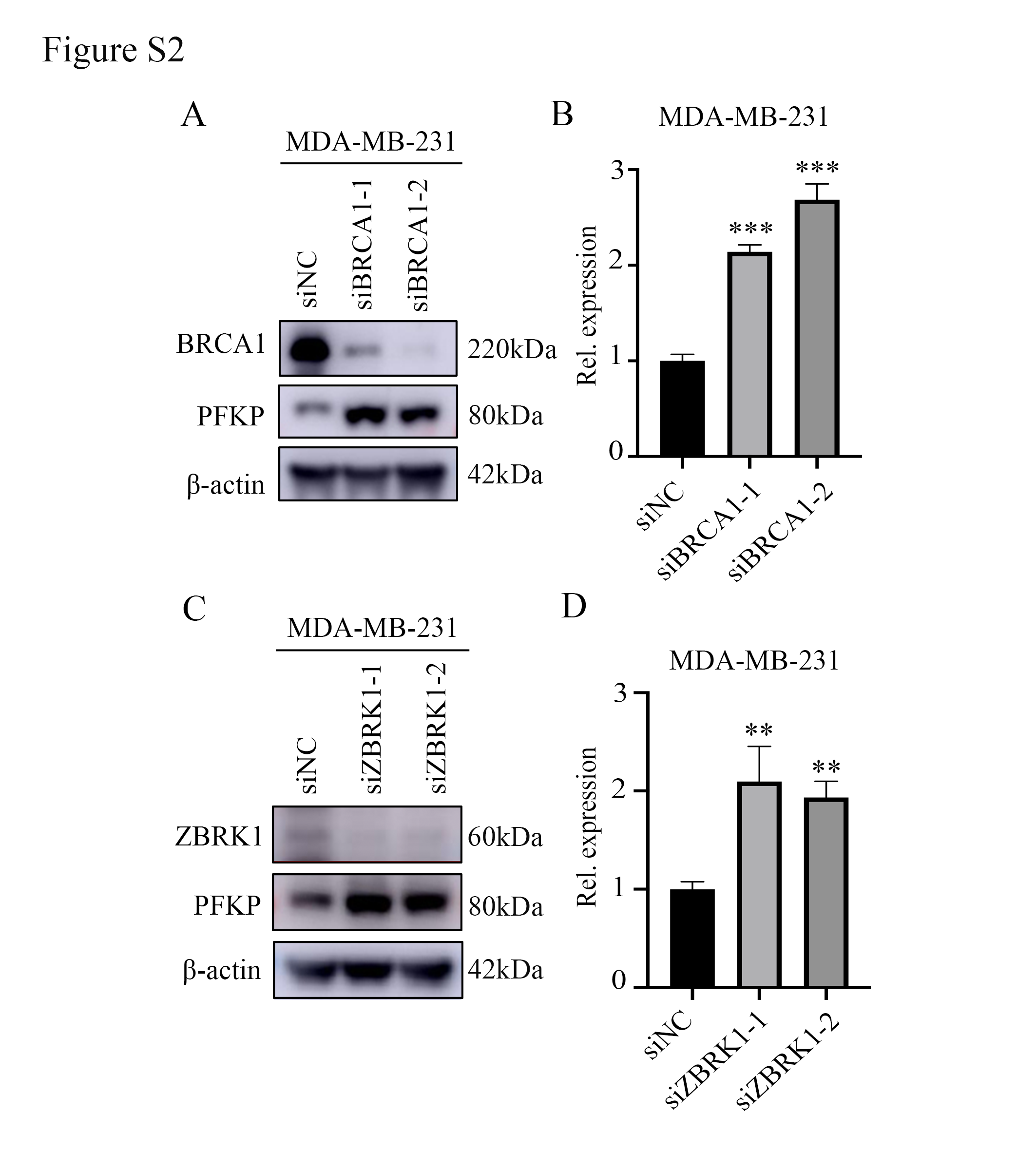

Supplement: S2 Fig — (A) Western blot detected the expression of PFKP in BRCA1 knocked down MDA-MB-231 cells and control counterparts. (B) Real time PCR detected the expression of PFKP in BRCA1 knocked down MDA-MB-231 cells and control counterparts. (C) Western blot examined the expression of PFKP in ZBRK1 knocked down MDA-MB-231 cells and control groups. (D) Real time PCR verified the expression of PFKP in ZBRK1 knocked down MDA-MB-231 cells and control groups. All experiments were biological replications. ***p < 0.001. **p < 0.01. (TIF) [file pone.0233750.s006.tif]

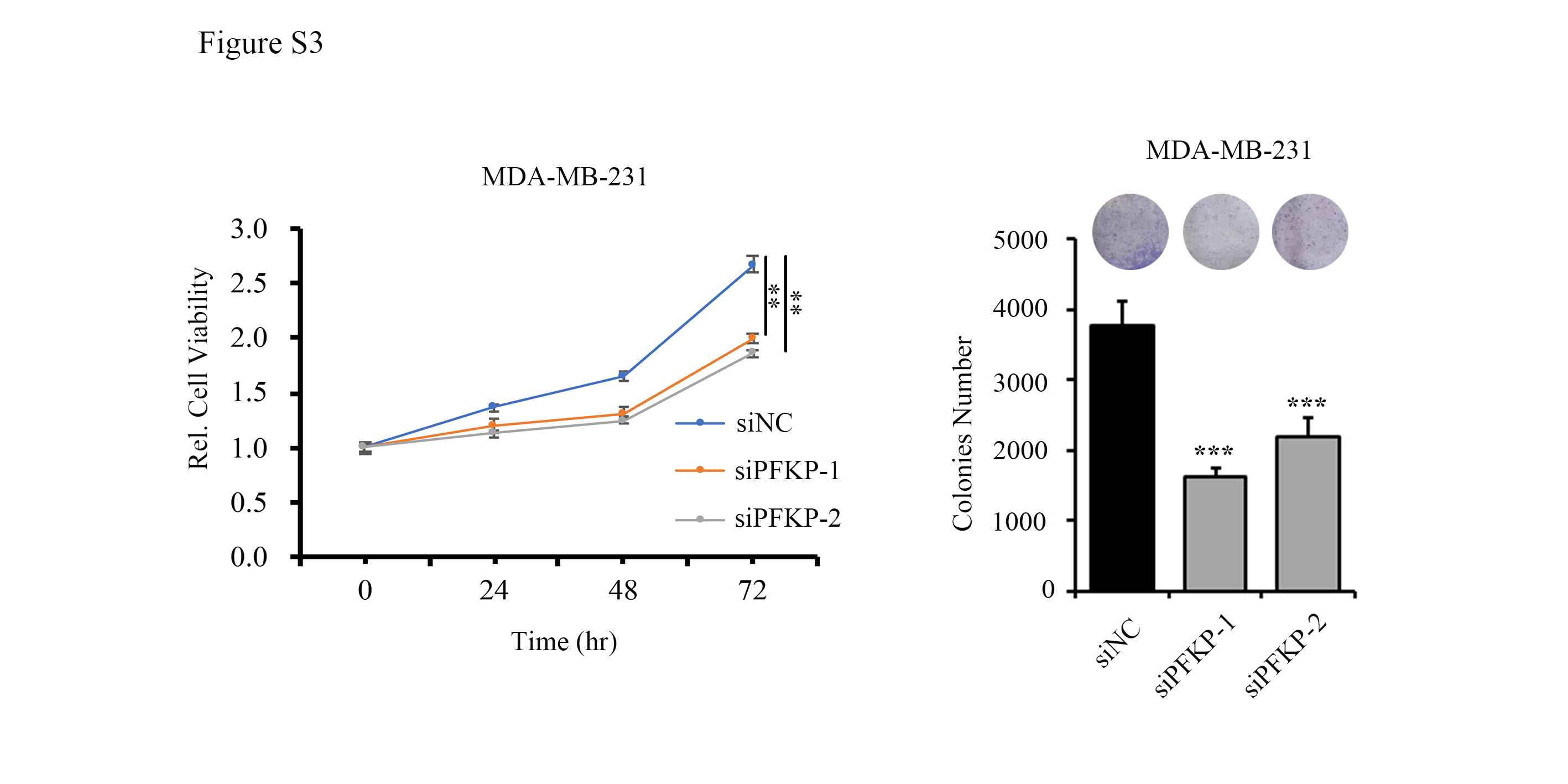

Supplement: S3 Fig — (A) MTT assay for growth curve of MDA-MB-231 cells. (B) Colony formation assay in MDA-MB-231 cells showed that PFKP sufficient cells had stronger growth energy. ***p < 0.001. **p < 0.01. (TIF) [file pone.0233750.s007.tif]

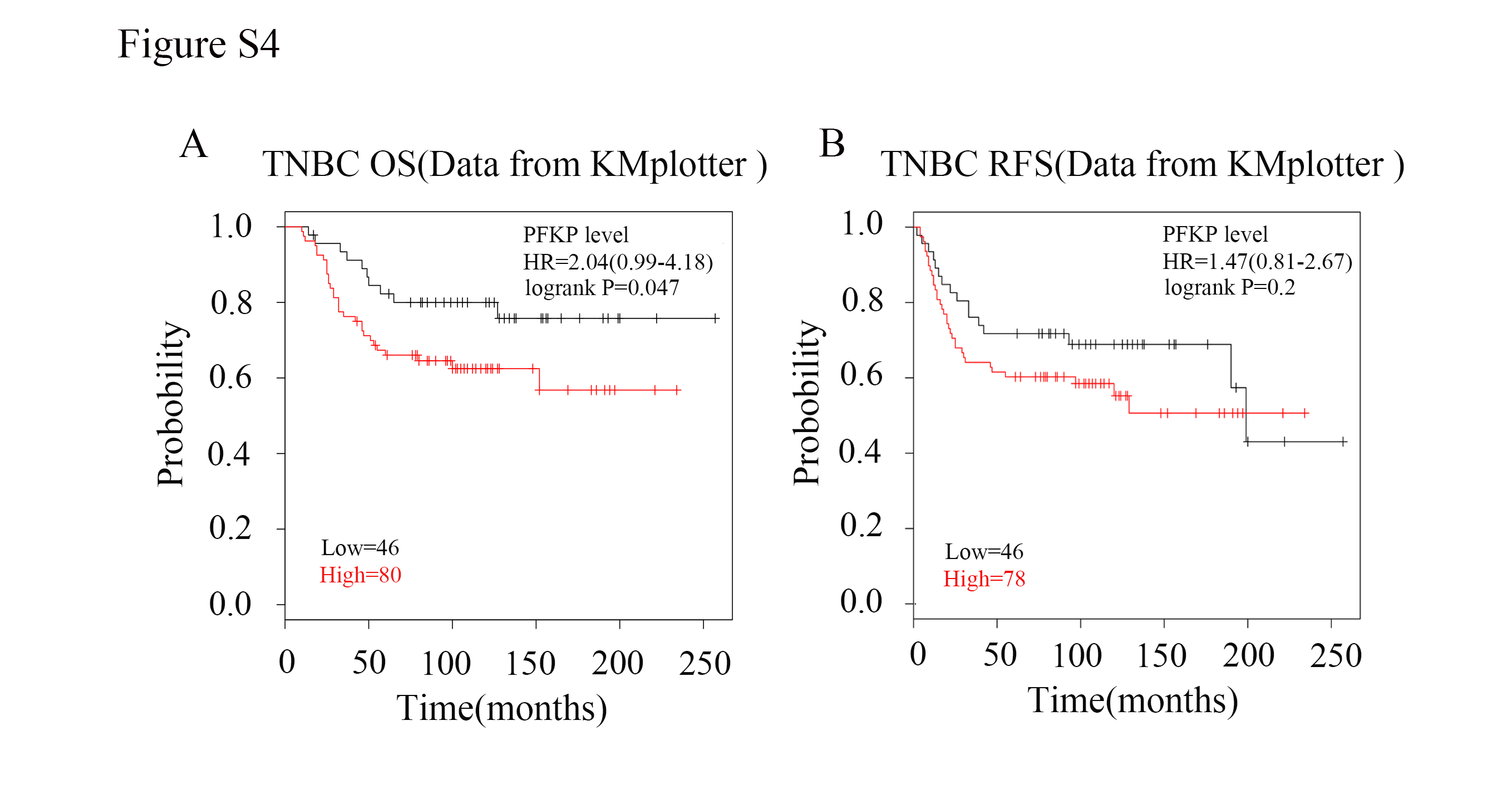

Supplement: S4 Fig — (A) KM plotter database showed that PFKP mRNA levels were closely related to overall survival in triple-negative breast cancer patients (OS, n = 126, P = 0.047). (B) KM plotter database showed that PFKP mRNA levels were not statistically corelated with triple-negative breast cancer patients' relapse free survival (RFS, n = 124, p = 0.2). (TIF) [file pone.0233750.s008.tif]

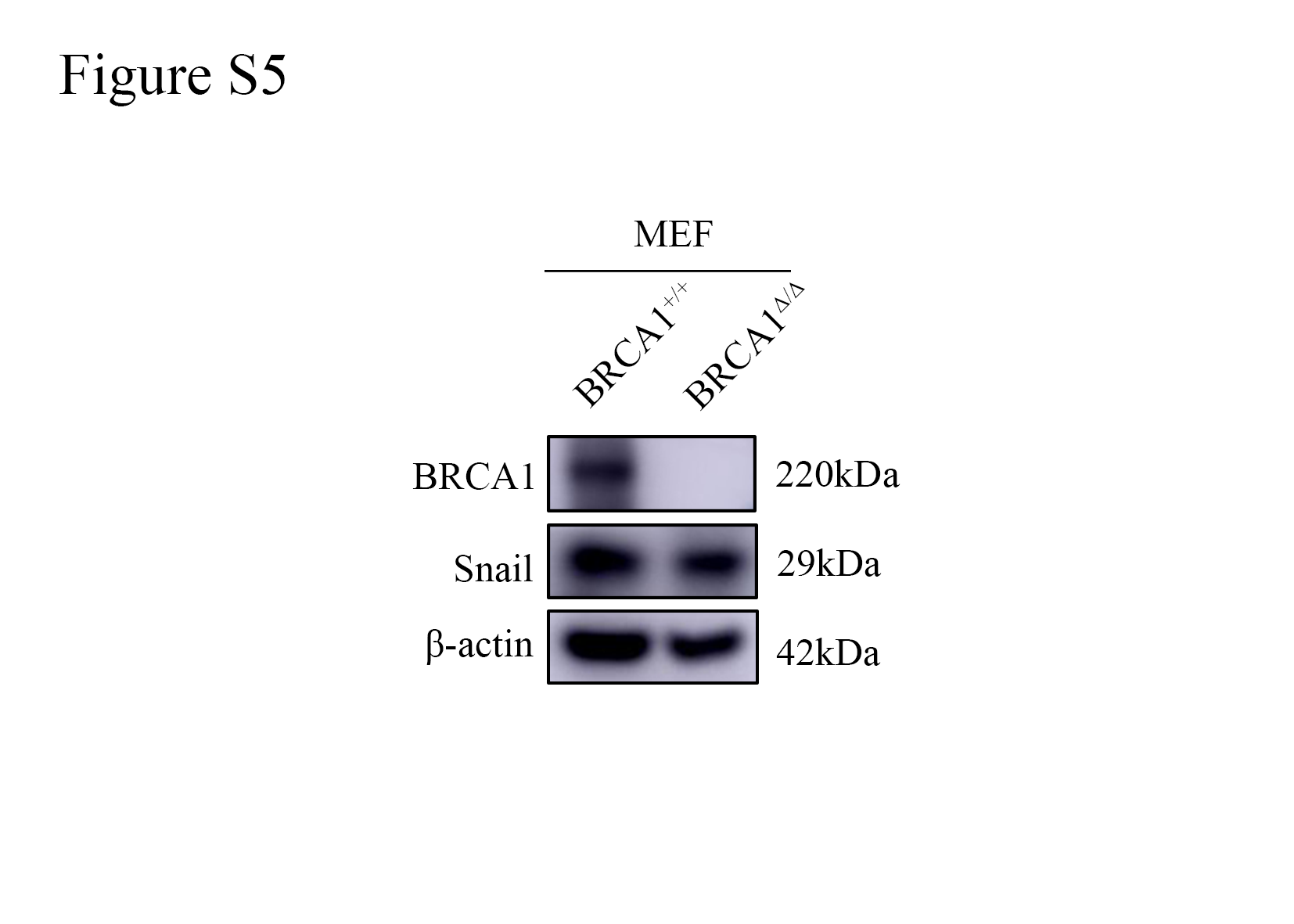

Supplement: S5 Fig — Western blot examined the expression level of Snail in MEF-BRCA1△/△ and MEF-BRCA1+/+ cells. (TIF) [file pone.0233750.s009.tif]
