# Supplementary material for: PFKP is transcriptionally repressed by BRCA1/ZBRK1 and predicts prognosis in breast cancer
Source: PLoS One. 2020 May 29;15(5):e0233750. doi: 10.1371/journal.pone.0233750 (PMC7259711; doi:10.1371/journal.pone.0233750)
Supplement: S1 Raw Images — (PDF) [file pone.0233750.s010.pdf]

Figure 1E

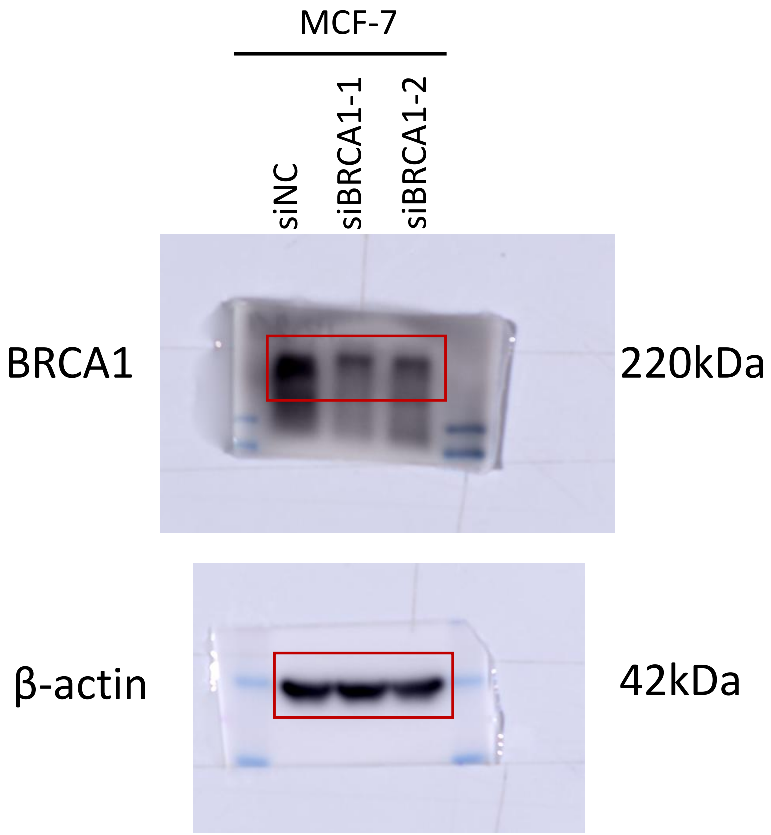

The chemiluminescence signals were detected with Amersham Imager 600 (GE, USA)

Figure 2F

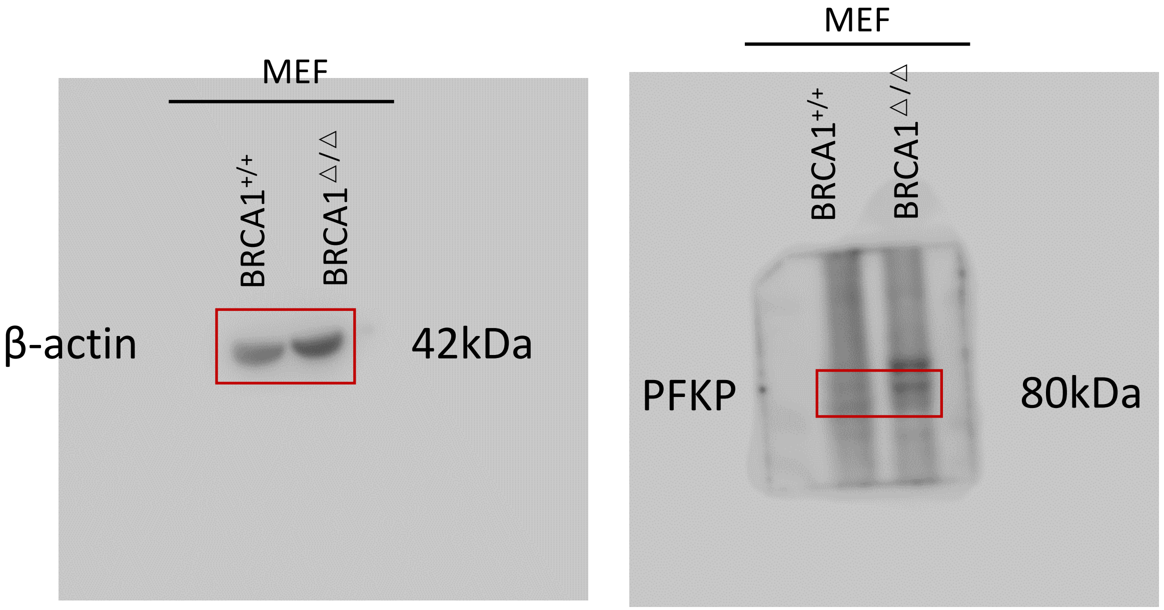

The chemiluminescence signals were detected with Amersham Imager 600 (GE, USA)

Figure 2G, H

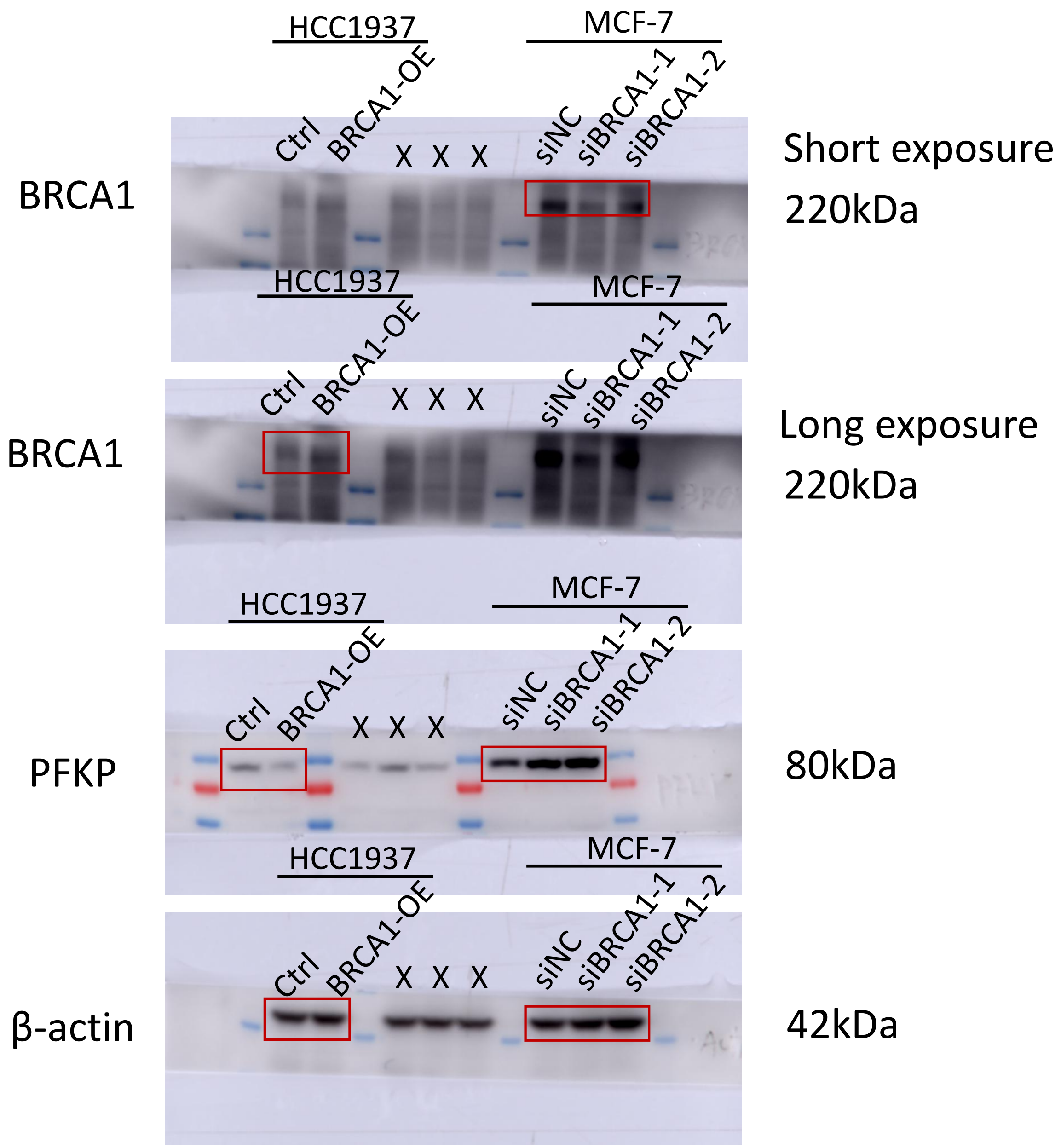

The chemiluminescence signals were detected with Amersham Imager 600 (GE, USA)

Figure 3B

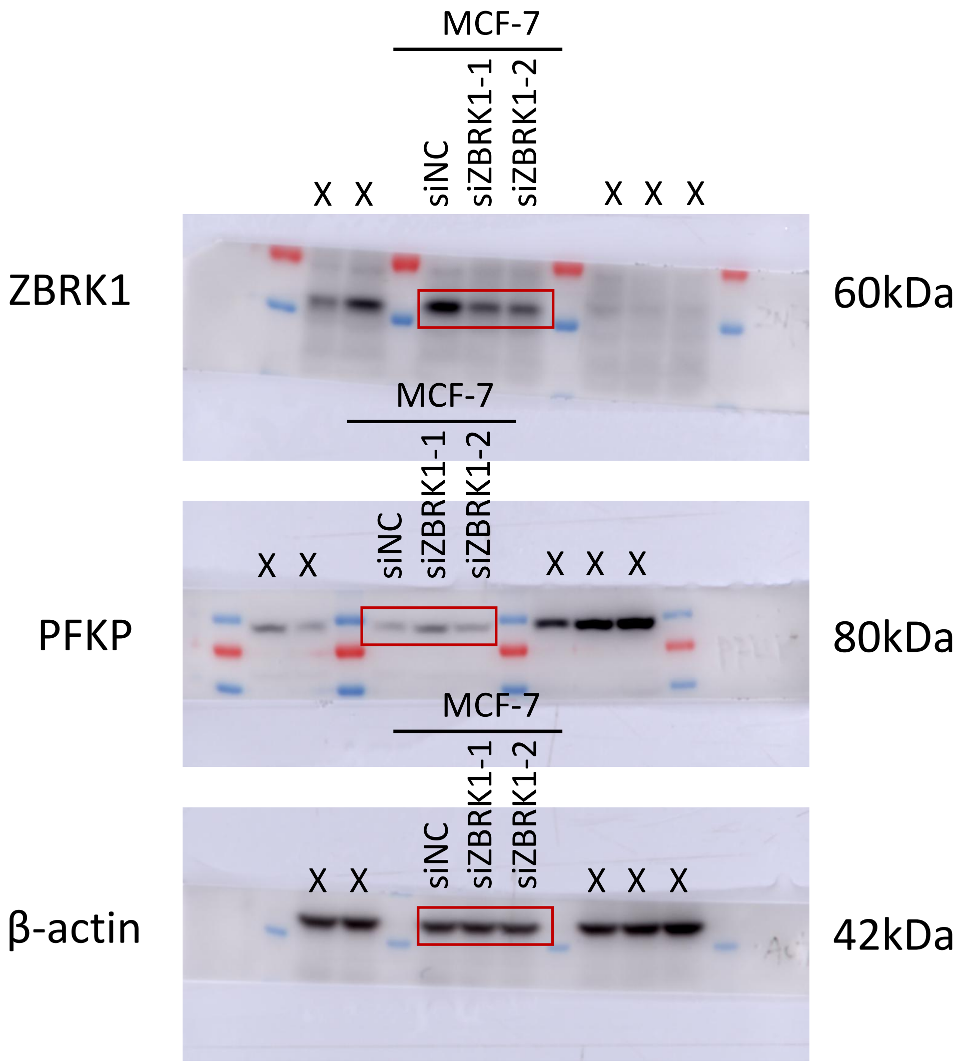

The chemiluminescence signals were detected with Amersham Imager 600 (GE, USA)

Figure 4D, E

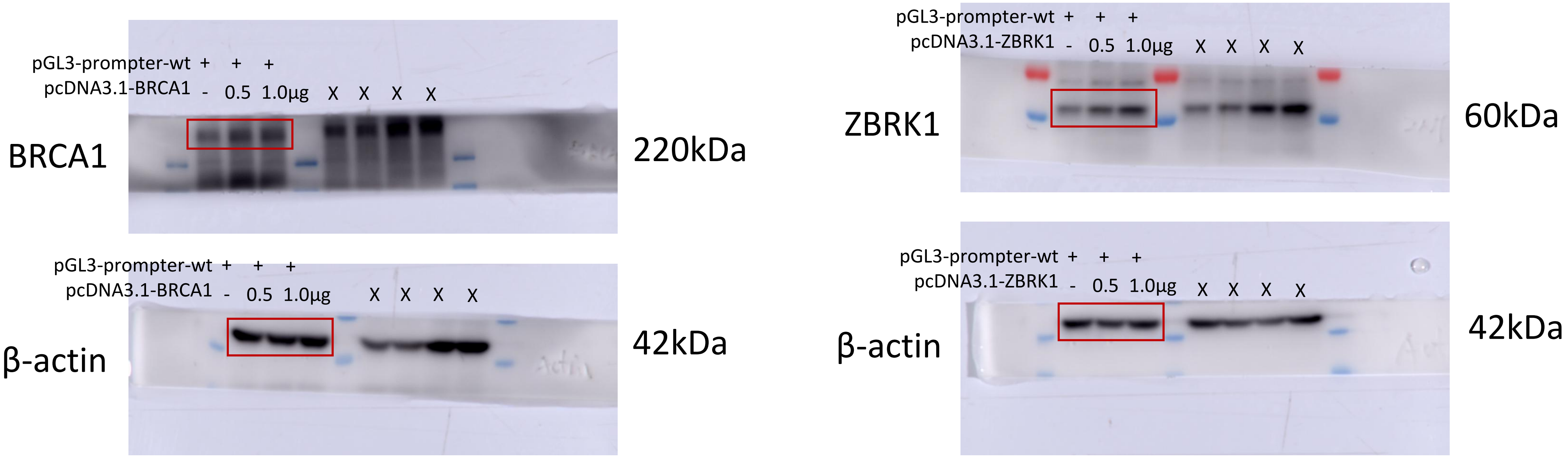

The chemiluminescence signals were detected with Amersham Imager 600 (GE, USA)

Figure S2A, C

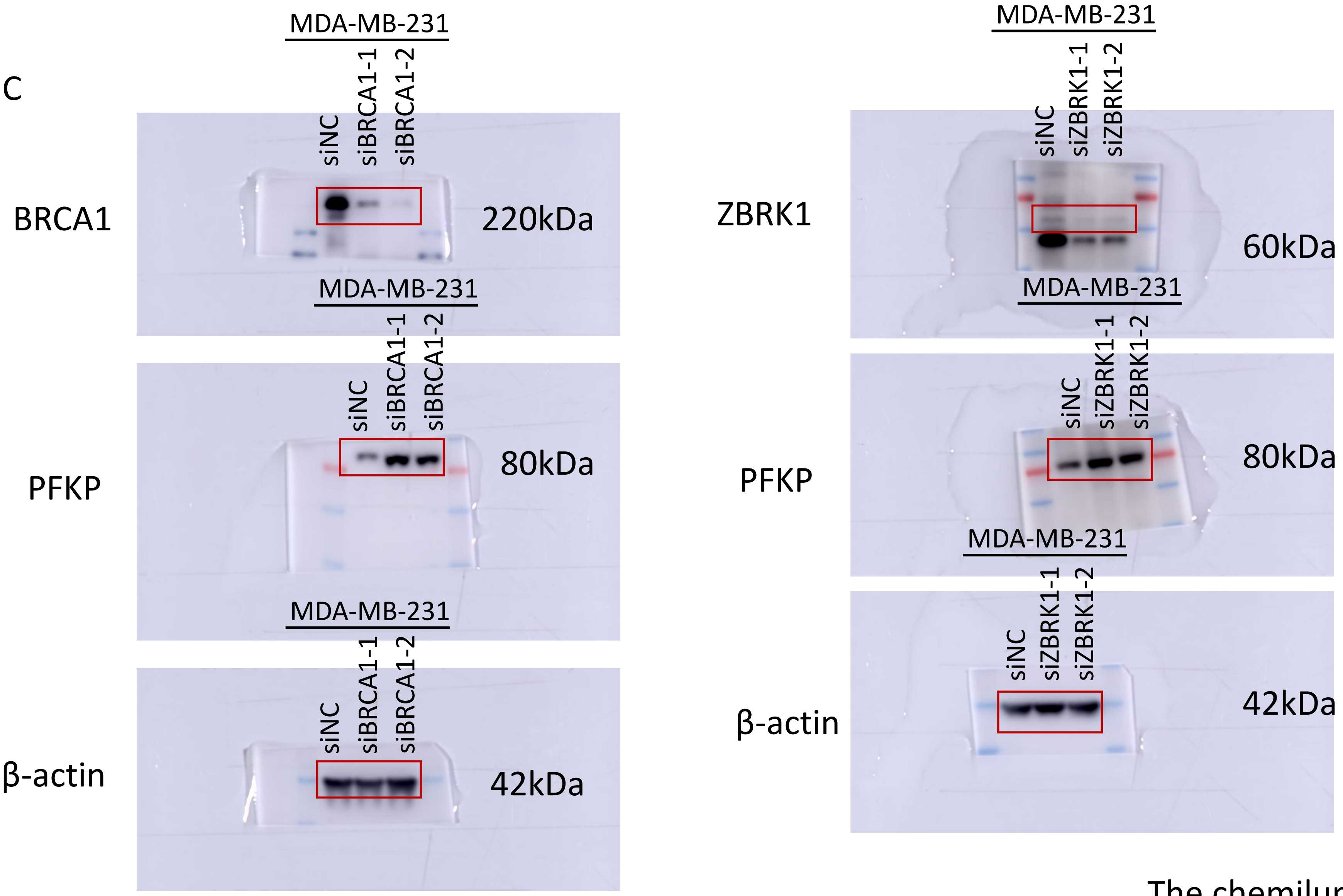

The chemiluminescence signals were detected with Amersham Imager 600 (GE, USA)

Figure S5

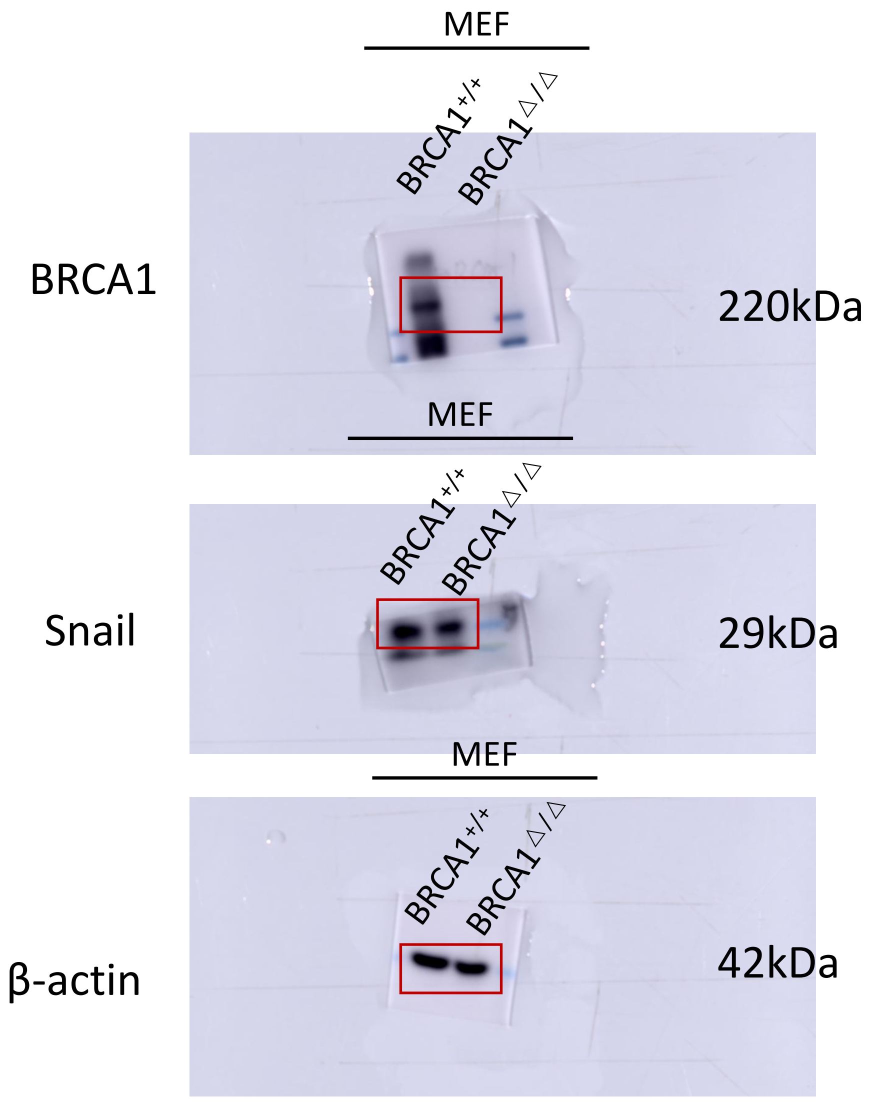

The chemiluminescence signals were detected with Amersham Imager 600 (GE, USA)
